# Nutritional assessment of institutionalized elderly

**DOI:** 10.1590/S1679-45082013000100007

**Published:** 2013

**Authors:** Milena Maffei Volpini, Vera Silvia Frangella

**Affiliations:** 1 Centro Universitário São Camilo - São Paulo, SP, Brazil

**Keywords:** Nutrition of the elderly, Health of the institutionalized elderly, Nutritional status, Long-stay institution for the elderly, Aging

## Abstract

**Objective:**

To define the nutritional profile of institutionalized elderly individuals.

**Methods:**

Comparative correlation and quantitative field study conducted in a Long-Stay Institution in Sao Paulo (SP), Brazil, between December 2010 and January 2012. To define nutritional diagnosis, data were collected from patient files, such as body mass index, circumferences, triceps skinfold, muscle area of the arm, thickness of the adductor pollicis, handgrip strength, and biochemical test results. The anthropometric variables were presented as mean, standard deviation, and percentages, and were grouped by gender and stratified by age. The level of statistical significance was p<0.05.

**Results:**

One hundred and two elderly individuals were selected, and 84 were females. Excess weight was the most common anthropometric diagnosis in men (n=11; 61%), with the detection of protein depletion in those aged 70 years, and possible cases of sarcopenic obesity. All women were in good health conditions (n=84; 100%). However, in 27% (n=23) of them, protein depletion was evident.

**Conclusion:**

More anthropometric studies are necessary which would allow a definition of local reference standards, stratified by gender and age group. The difference between populations and factors, such as inclusion and exclusion criteria, and methodological characteristics, limit the use of international standards, interfering in the reliability of the nutritional diagnosis.

## RESUMO

**Objetivo:**

Definir o perfil nutricional de idosos institucionalizados.

**Métodos:**

Estudo de campo comparativo correlacional e quantitativo realizado em uma Instituição de Longa Permanência, em São Paulo (SP), entre dezembro de 2010 a janeiro de 2012. Para definição do diagnóstico nutricional, coletaram-se dados de prontuários acerca de índice de massa corporal, circunferências, dobra cutânea tricipital, área muscular do braço, espessura do músculo adutor do polegar, força de preensão palmar e resultados das provas bioquímicas. As variáveis antropométricas foram apresentadas como média, desvio padrão e percentis, sendo agrupadas segundo o gênero e estratificadas por idade. O nível de significância estatística foi de p<0,05.

**Resultados:**

Selecionaram-se 102 idosos, sendo 84 do gênero feminino. Excesso de peso foi o diagnóstico antropométrico que mais ocorreu nos homens (n=11; 61%) detectando-se depleção proteica a partir dos 70 anos e possíveis casos de obesidade sarcopênica. Todas as mulheres apresentaram-se eutróficas (n=84; 100%). Entretanto, em 27% (n=23) delas, evidenciou-se a depleção proteica.

**Conclusão:**

São necessários outros estudos antropométricos que permitam definir padrões de referências locais, estratificados por gênero e faixa etária. A diferença entre populações e fatores, como critérios de inclusão e exclusão e características metodológicas, limitam a utilização de padrões internacionais, interferindo na confiabilidade do diagnóstico nutricional.

## INTRODUCTION

The increase in the elderly population is a universal phenomenon. Currently, in Brazil, the number of individuals aged 60 years or more, corresponds to 18 million, representing 12% of the total population, i.e., almost 5% more than that observed in the 2001 Census, which showed 7.3%. Recently, an increase of the population 80 years of age and older has also been observed, which today represents 3 million of the total number of elderly persons in the country^(1)^.

Together with this scenario, changes in family structure and society dynamics which have occurred ever more frequently, with a growing insertion of women in the work market, explain the fact that many families choose to institutionalize their elderly^(2)^. However, this institutionalization imposes changes in the daily routine of these individuals, including feeding, and can generate alterations in eating habits and fragility of their health, due to less acceptance of food with the consequent compromise of their nutritional status^(3)^.

The lack of early diagnosis of malnutrition in the elderly may be reflected in deterioration of health and increased risk of mortality^(4)^. In this way, nutritional evaluation may help professionals in treating for recovery and promotion of health in the elderly^(4)^. Anthropometric studies commonly use the parameters of body mass index (BMI), circumferences of the arm (AC) and of the calf (CC), circumference and muscular area of the arm (AMC and AMA), where values are evaluated as per international reference standards, such as those by Burr and Phillips^(5)^.

## OBJECTIVE

To define the nutritional profile of institutionalized elderly individuals, analyzing the application of anthropometric indicators and biochemical tests, as well as to understand the limitations of these parameters for diagnosis of malnutrition in the elderly and to propose more sensitive ones for the population studied.

## METHODS

This is a cross-sectional descriptive study conducted in a long-stay institution (LSI), located in the neighborhood of Butanta, in Sao Paulo City (SP), where 196 elderly of both genders lived, and 151 (77%) of them were females.

Inclusion criteria adopted were elderly residents at the institution who would accept and collaborate in the collection of anthropometric data and whose medical records contained the results of the following laboratory tests requested by the physicians: hematocrit (Ht), hemoglobin (Hb), total leukocytes and lymphocytes (to calculate the total lymphocyte count TLC), total serum proteins, albuminemia, and cholesterolemia. Therefore, of the total number of institutionalized residents, 94 (48%) were excluded, since 81 (41%) had no record in their charts of all biochemical tests, 10 (5%) refused to participate in the study, and 3 (2%) presented with aggressive behavior at the time of anthropometric measure assessment.

Definition of the sample was made randomly, and not probabilistically.

Data collection was performed after approval of the Research Ethics Committee, under number 15/011.

The following recorded data were collected from the medical records: age, medical diagnosis, time of institutionalization, feeding route, consistency of the diet offered, and degree of dependence (classified as per Functional Independence Measure –FIM)^(6)^.

To define the nutritional diagnosis, BMI, AC, AMC, AMA, CC, triceps skinfold (TSF), thickness of the adductor of the pollaris muscle (TAPM), and palm grip force (PGF) were used. These measurements were obtained in accordance with techniques established in the literature^(5,7–11)^.

The classification proposed by the Nutrition Screening Initiative (NSI)^(12)^ was used for evaluation of the values of BMI. The results of TSF, AMC and AMA were compared to those percentiles proposed by Burr and Phillips^(5)^. To evaluate the results of the TSF, the following standards were adopted: reduced adipose reserve (≤ percentile 25); good general conditions (> percentile 25 and < percentile 75), and excess adipose reserve (≥ percentile 75). For the values of AC, AMC and AMA, the following parameters were adopted: good general conditions (> percentile 25) and malnutrition (≤ percentile 25)^(13)^.

CC was considered adequate when it showed values ≥31cm for both genders, as proposed by Vellas et al.^(14)^.

For the classification of TAPM, the criterion established by Lameu et al. was used^(7)^.

The classification of PGF measurement results followed that proposed by Barbosa et al.^(15)^: score 0 (percentile ≤10) = incapable; score 1 (percentile ≤25) = weak; score 2 (< percentile 25 and ≤ percentile 75) = moderate; score 3 (> percentile 75) = very good.

The results of the laboratory tests were collected and evaluated as well, using as reference values for hemoglobin and hematocrit those proposed by Bottoni et al.^(16)^. Calculation of the total lymphocyte count also followed that proposed by Bottoni et al.^(16)^, considering as mild depletion results with values of 1,200 to 2,000/mm^3^; moderate depletion as values of 800 to 1,199/mm^3^; and severe depletion with values <800/mm.

As normality, the value of 6.4 to 8.1g/dL was adopted for analysis of the results of the total serum protein^(16)^. The values of albuminemia were interpreted in the following way: normal >3.5g/dL; mild depletion: 3-3.5g/dL; moderate depletion: 2.4-2.9g/dL; severe depletion: <2.4g/dL^(16)^.

Cholesterol levels of < 160mg/dL were considered indicators of malnutrition^(17)^.

Statistical analysis was made using the Student's *t* test and Pearson's linear correlation coefficient. For this, Microsoft Excel 2007 and Statistical Package for the Social Science (SPSS), version 15.0 were used, adopting as significant values of p<0.05.

## RESULTS

Thus, 102 elderly composed the sample, representing 52% of the total population, with a mean age of 84.9±7.8 years (minimum of 60 years and 1 month and maximum of 104 years and 5 months). Of this total, 18 elderly individuals were males (17.6%) and 84 were females (82.4%), with mean ages of 83.2±8.52 and 86.2±7.6 years, respectively.

Among the elderly, 61% (n=11) of the male group and 82% (n=69) of the female group were aged ≥ 80 years, in which the nonagenarians and centenarians represented 29% (n=28) and 3% (n=3) of this total, respectively.

The mean time of institutionalization was 2.74±3.3 years for the male group and 6.8±7.4 years for the female group.

It was further noted that 44% of the men (n=8) received a soft diet; 33% (n=6) light diet; 17% (n=3) enteral, and only 6% (n=1) had a general diet. Whereas in the female group, 46% (n=39) received a general diet, followed by 28% (n=23) soft; 18% (n=15) light, and 8% (n=7) enteral.

Among the medical diagnoses, Alzheimer's disease (AD) was the one most often identified in both genders, with 39% (n=7) occurrence in males (66.6% in cases of advanced stage) and 46% in females (60% in advanced stage). In the male group, this diagnosis was followed by diabetes mellitus and then by cardiovascular diseases (coronary disease, atherosclerosis, congestive heart failure, and myocardial sclerosis) and, finally, by Parkinson's disease. In the female population, on the other hand, the most frequent disorder after types of dementia was arterial hypertension (32% of cases; n=27), followed by hypothyroidism, diabetes mellitus, and dyslipidemia.

The results presented in table 1 show that the mean BMI was 24.6±2.2kg/m^2^ (minimum of 17.64kg/m^2^ and maximum of 34.32kg/m^2^) in the male group, and 23.9±3.5 kg/m^2^ in the female group (minimum of 15.23 kg/m^2^ and maximum of 38.6 kg/m^2^), characterizing the sample as eutrophic, in which the male group presented with higher values. Also noted was that in the male group, over 95 years of age, and in females, over 100 years of age, the values of BMI were lower than the reference value proposed (17.7 and 21.4±1.5kg/m^2^, respectively). On the other hand, the mean values of AMC and AMA in the male group were superior to those of the female group for all age groups, and these differences proved statistically significant (p=0.003 and p=0.013, respectively). Also noted was the reduction of the values of AMC and AMA, for both genders, which was only expressive over 90 years of age. The mean TSF in men was 12.6±2.3mm, and in women it was 16.5±5.0mm, indicating that the female gender presented greater adipose reserve. Only 60% of the elderly (n=59) presented with values appropriate for the measurement of CC (Table 1).

**Table 1:** Anthropometric results of the elderly

Anthropometric data	Male - age (years)								
	60-65	70-75	75-80	80-85	85-90	90-95	95-100	100 or more	Mean
n=18	1	1	5	2	2	6	1	0	
BMI	26.9	27.5	27.2±3.6	25.2±3.1	23.1±0.1	24.7±1.9	17.7	–	24.6±2.2
AC	29	26.5	28.9±1.8	29.7±32	28.1±1.3	28.7±1.7	23.3	–	27.7±2.0
AMC	25.5	23	24±2.5	245±3.5	245±0.6	24.1±2.2	20.49	–	23.7±2.2
AMA	51.9	42.3	46.2±9.0	48.3±13.5	56.5±14.7	46.5±9	33.4	–	46.4±11.5
TSF	11	11	15.3±2.6	16.5±0.7	11.5±2.1	13.9±4.0	9	–	12.6±2.35
Final diagnosis	Eutrophic	Eutrophic	Excess weight	Excess weight	Excess weight	Excess weight	Eutrophic		
**Anthropometric data**	**Female - age (years)**								
	**60-65**	**70-75**	**75-80**	**80-85**	**85-90**	**90-95**	**95-100**	**100 or more**	**Mean**
**n=84**	**2**	**3**	**10**	**13**	**30**	**15**	**8**	**3**	
BMI	23.4±0.7	23.2±3.9	252±6.2	25.5±5.0	23.7±4.5	245±3.6	24.2±3.0	21.4±1.5	23.9±3.5
AC	29.2±1.2	26.5±46	282±6.0	27.1±3.5	25.8±3.3	262±2.5	24.1±2.0	243±2.9	26.4±3.2
AMC	21.4±0.3	20.8±45	23.1±4.2	21.1±2.5	21.3±2.9	21.7±1.5	20.1±1.8	20.2±2.2	212±2.5
AMA	33.2±4.3	36.1±15	43.9±16.3	35.8±8.6	36.7±9.9	37.6±5.2	32.6±6.06	33.0±7.2	36.1±9.1
TSF	25.5±5	15.1±4.4	16.1±6.6	19.1±6.1	14.6±5.3	14.3±4.6	14.4±4.5	13±3.5	16.5±5.0
Final diagnosis	Eutrophic	Eutrophic	Eutrophic	Eutrophic	Eutrophic	Eutrophic	Eutrophic	Eutrophic	

BMI: body mass index; AC: arm circumference; AMC: arm muscle circumference; AMA: arm muscle area; TSF: triceps skinfold

It was also observed that, over 75 years and up to 95 years of age in men, the final nutritional diagnosis of excess weight was more frequent, while in women, the diagnosis of good general conditions corresponded to 100% of the sample (Table 1).

Table 2 presents the results of TAPM and PGF measurements, as per gender and age group. The mean values of TAPM in the male group, except for the age groups of 70 to 75 years and from 95 to 100 years, revealed that the elderly were in good general conditions. In the female group it was noted that, except for the age group of 70-75 years, the results were within normality (9.55±2.0mm).

**Table 2:** Result of anthropometrics as per the calf circumference (CC), thickness of the adductor pollicis muscle (TAPM) and palm grip force (PGF)

Anthropometric data	Male - age (years)							
	60-65	70-75	75-80	80-85	85-90	90-95	95-100	100 or more
n=17	1	1	5	2	2	5	1	–
CC	35	32	34±4.2	348±2.6	349±2.9	30.2±2.0	23	–
TAPM R	12	9	11.1±1.3	15±4.2	12±1.4	9.8±6.7	7	–
**n=8**	**1**	**–**	**2**	**1**	**2**	**2**		
PGF R	16	–	8.7±0.7	0	0	5.5±7.8	–	–
PGF L	17	–	4.5±4.5	0	0.75±1.1	4.0±4.2	–	–
Diagnosis final	Depletion of protein	Depletion of protein	Depletion of protein	Depletion of protein	Depletion of protein	Depletion of protein	Depletion of protein	Depletion of protein
**Anthropometric data**	**Female - age (years)**							
	**60-65**	**70-75**	**75-80**	**80-85**	**85-90**	**90-95**	**95-100**	**100 or more**
**n=84**	**2**	**3**	**10**	**13**	**30**	**15**	**8**	**3**
CC	35.2±5.7	34.5±5.1	31±4.5	32.9±44	31.5±41	31.0±2.9	296±5.4	30.8±0.8
TAPM R	9.5±0.5	7±1.6	10.6±3.0	11.5±2.6	10±2.4	10.7±1.9	8.4±2.2	8.7±1.8
**n=37**	**1**	**1**	**4**	**6**	**12**	**8**	**3**	**2**
PGF R	0	8	3.1±3.1	8.7±1.9	2.6±3.7	3.0±3.0	0	0
PGF L	4	0	5.1±5.0	7.4±2.0	2.6±3.8	3.2±3.2	0	0.25±0.25
Diagnosis final	Depletion of protein	Depletion of protein	Depletion of protein	Depletion of protein	Depletion of protein	Depletion of protein	Depletion of protein	Depletion of protein

R: right; L: left

There was statistical significance between TAPM and AC (p=0.016), and TAPM and AMC (p=0.033) only in the male group, since in females it corresponded to p=0.357 and p=0.139, respectively.

In the evaluation of PGF, excluded from the sample were the elderly who were not in cognitive condition to understand the instructions for their mensuration. Thus, the measurement was made only in 44.1% (n=45) of the sample, 82% of it in females (n=37). In the male gender, for all age groups, the classification was of “incapable”, as well as in the female gender, with the exception of women aged 80-85 years, on the right side, whose score was one, resulting in the classification as “weak”. However, it was noted that the mean PGF value in the male group, for both sides, was superior relative to that of the women (PGFr=6.04kg and PGFl= 5.25 kg versus PGFr =3.17kg and PGF=2.81kg, respectively). Statistical analysis revealed non significance and a weak correlation between the PGF and the evolution parameters of lean mass for both genders, except between TAPM and PGFl and only in the female gender.

As can be seen on table 3, for the male gender, the hematocrit was reduced for all age groups, as well as hemoglobin, over 80 years of age (value = 11.7±2.8g/mL). Whereas in the female gender, the result of the hematocrit was also reduced over 70 years of age (37.7±2.0%) and for hemoglobin it was reduced only between 90 and 100 years of age (11.7±1.3g/mL). The total count of lymphocytes proved reduced in 62% of the elderly (n=63), and of these, 78% (n=49) were classified as having protein depletion. There was a good correlation of the total lymphocyte count and the results of anthropometric measurements (p=0.05).

**Table 3:** Results according to the laboratory tests

Laboratory tests	With depletion		Without depletion	
	M	F	M	F
	n=5 (28%)	n=32 (38%)	n=13 (72%)	n=52 (62%)
Hematocrit	38.28±8.3	34.9±9.3	38.65±4.1	36.9±3.8
Hemoglobin	12.92±2.5	11.4±3.1	13.07±1.4	12.0±1.4
TLC	1512±515.6	1823.1±10.2	1651±569.7	1880.8±648.2
Total protein	6.02±0.5	5.8±1.6	6.4±0.4	6.3±0.6
Albumin	3.6±0.1	3.4±0.9	3.6±0.5	3.6±0.55
Total cholesterol	179.8±33.5	165.3±50.1	154.1±21.9	177.9±37.16

TLC: total lymphocyte count

The total protein values of the male group were reduced in all age groups older than 70 years (except from 80 to 85 years of age). In females, however, these values were reduced in all age groups over 75 years (except in the group of 75 to 80 years), with a mean value of 6.3±0.34g/dL. Among the elderly of the sample, 64% (n=63) presented with total protein values below what is recommended, indicating protein depletion. Nevertheless, this diagnosis was not shown in the anthropometric measurements.

Hypoalbuminemia was identified in males in the age groups 60-65 years (2.74g/dL) and 80-90 years (3.2±084g/dL), results that are not related to the final anthropometric diagnosis, since for these age groups, the diagnoses found were of good general conditions and excess weight, respectively. In females, no reduced values were found for this test, and the statistical analysis suggested a good correlation (p=0.05) with the other anthropometric measurements, which diagnosed good general conditions in all age groups analyzed.

Hypocholesterolemia was found in males for all age groups over 75 years, which showed depletion of lean mass detected only by PGF. On the other hand, the female group presented no hypocholesterolemia in any age groups.

The result of the biochemical analysis and of the anthropometric evaluation suggest protein depletion in males for the age group 70-75 years, evidenced by the reduction in values of total protein and of TAPM from 75 to 80 years, and from 85 to 90 years of age, identified by the serum values for total protein, albumin, and cholesterol. Nevertheless, the result of the anthropometric evaluation diagnosed these elderly individuals as having excess weight. Thus, the evaluation of all parameters studied suggests as final nutritional diagnosis the classification of these individuals of the male gender as probable sarcopenic obesity.

## DISCUSSION

The mean time of institutionalization found in the present study differed from that obtained in the study carried out by Guedes and Silveira^(18)^, with 109 elderly persons from the city of Passo Fundo (RS), which was 7.99 years. It was also noted that the women were institutionalized for a longer time. This result might be explained, in part, by the fact that women live longer, become widows earlier, and display greater difficulty in marrying or remarrying after a separation or widowhood ^(19)^.

Alterations in the consistency of the diet may be related to structural, morphological, and biochemical modifications typical of aging, such as the change in muscle composition, reduced salivation and dental problems, and use of maladapted dental prostheses. We point out, however, that the modification in the consistency of the diet in very elderly individuals also is generally necessary due to difficulty in swallowing and/or the risk of aspiration of food. In the elderly, the incidence of dysphagia is high and appears as a symptom of some diseases, such as cerebrovascular accidents (CVA), Parkinson's, and Alzheimer's, diseases that frequently affect this population^(20)^, which can also be observed in the present study. Alzheimer's disease (AD), which was the medical diagnosis most often found, represents 50 to 60% of cases of dementia, affecting approximately 1% of the Brazilian population and 10 to 20% of elderly individuals, doubling in prevalence every 5 years, over 65 years of age. Since it is characterized by a deficit in memory and in other cognitive functions, Alzheimer's disease causes deterioration in functional capacity, making the individual clearly incapable of performing activities of daily life (ADLs), and depending on caregivers, which could justify the high incidence of dependency found in most studies^(19)^.

The participants of the male group presented a more compromised degree of dependency than the females. Advanced age may be one of the factors that justifies such results^(20)^, since the chance of an elderly person presenting moderate to serious dependency is approximately 36 times greater for those over 80 years of age, which corroborates the research data. The results also reaffirm the fact that institutionalization is, in most cases, associated with greater physical and cognitive dependency^(21)^.

The results of BMI are similar to those of national studies developed with institutionalized elderly individuals, such as that carried out by Felix and Souza^(22)^, in the Federal District, with 37 elderly persons institutionalized, in which the mean BMI in men was 25.4kg/m^2^ and of 23.8kg/m^2^ in the female group; but superior to what was found by Menezes and Marucci^(9)^ when evaluating 305 elderly individuals from LSIs in Fortaleza (CE), where the BMI was 22.4±4.6kg/m^2^ in men and 23.0±5.3kg/m^2^ in women. The tendency was noted towards a decrease in BMI for the higher age groups, as has been reported in other studies already published. Despite this tendency, the mean values are unique, possibly due to the differences in race and socioeconomic levels. This confirms the need for local reference standards for each age group^(13)^.

The results found in AMC and AMA corroborate those of other studies, such as those performed by Felix and Souza^(22)^, and that developed by Cardoso^(3)^, who evaluated 53 elderly institutionalized residents in the southern part of the state of Minas Gerais. In all these studies, the mean values of AMC and AMA were greater in the male group, for all age groups, indicating that men have more muscle mass than do women in all age groups. Nevertheless, the reduction of these values only over 90 years of age in both genders differs from the results found by Menezes and Marucci^(13)^ and by Perissinotto et al.^(8)^, who found a tendency towards a decline in muscle mass both in men and in women over 65 years. Such a result might suggest better nutritional care as to the adjustment of the food provided for the elderly we evaluated, since there was a dietitian exclusive for the clinical area. We must point out that the reduction of AMC and the body weight loss are important indicators of the presence of malnutrition in the elderly^(8)^.

As to TSF, in the study performed in Florianopolis (SC) by Rauen et al.^(23)^ with 167 elderly institutionalized individuals, the mean values found were superior in the female group relative to the male group (15.5 *versus* 9.6mm). Thus, the mean found in the female group was similar to that of the present study, but in men it was inferior. Whereas in the study performed by Menezes and Marucci^(9)^, with 385 elderly residents in Fortaleza, the mean of 13 mm was observed in males, similar to that of the present study, and in women it was superior (21.3mm). Despite mean values being different, the results shown here are congruent with the information available in literature that indicates a greater accumulation of subcutaneous fat in women when compared to men^(3)^. The diagnosis of excess weight in the male group evaluated deserves attention since it reflects risks of chronic diseases, primarily cardiovascular disorders, diabetes mellitus, and arterial hypertension – diseases found in the sample evaluated. Other studies, such as that carried out by Felix and Souza^(22)^, also reported the occurrence of overweight (27% in a sample of 37 elderly persons). However, these results differ from literature, which points out that the highest frequency of overweight or obesity is seen among women, possibly explained by the fact that they have greater accumulation of visceral fat and greater life expectancy^(8)^. However, there is controversy as to the significance of excess weight among the elderly and its impact on their health, which seems to be less than that observed for adults, including in mortality rates. A study performed by Grabowski and Ellis^(24)^ with 7,527 elderly Americans analyzed the association between obesity and mortality and verified that this condition, compared to thinness and maintenance of weight within the normal weight range, may be protective for the occurrence of mortality in this population.

The results of good health conditions within the female group, in this study, differ from the value found in the study by Rauen et al.^(23)^, in which of the 135 elderly women studied, only 33.3% were in good general conditions. Such results may be explained by the fact of the institution having a nutrition team that draws up the weekly menu, accompanies acceptance of the food by the elderly, and carries out periodic nutritional evaluations with individual interventions whenever necessary.

The decline in calf circumference occurred in the oldest age groups, i.e., in men over 90 years and in women over 95 years. However, it is important to point out that a large part of the elderly evaluated were wheelchair-bound, generating grater reduction in their activities and producing, as a consequence, atrophy in the lower limbs. Kasper et al.^(25)^ further demonstrated that atrophy by lack of use starts at the fourth hour of rest in bed or even in a wheelchair, resulting in decreased muscle mass, diameter of the muscle cell, and number of muscle fibers.

Despite the values found in TAPM being within the range of normality, values superior to those of the present investigation were obtained in a study performed in Rio Grande do Sul, by Cabrera et al.^(11)^ in 48 elderly institutionalized persons, with a mean of 18.59±3.95mm. In face of the divergence from the results found, the difference between the age groups of the study participants is suggested as a relevant factor, since the mean age of the elderly in the research cited above was 71.3±8.17 years for males, while that of this study was greater. No studies were found evaluating TAPM in institutionalized elderly women for comparison. However, since this parameter is still seldom applied and tested in the practice of population studies and primarily, in institutionalized elderly individuals, caution is recommended in the use and interpretation of this measure, suggesting that it is advisable to always align it with other anthropometric and biochemical parameters.

Results of greater values of PGF in the dominant hand than in the non-dominant hand corroborate the values found by Souza et al.^(10)^, in which 241 children and adolescents, aged between 6 and 14 years, were evaluated also using the Bulb^®^ dynamometer (26.4 and 24.6kg, respectively). Even when the results herein described are compared with those of the studies that use the Jamar^®^ dynamometer, it is evident that the values of the dominant hand are greater. The differences between genders in neuromuscular activation, alterations in muscle temperature induced by hormones, differences in blood flow resulting from changes in mechanical compression, as well as the muscle size and use of substrates dependent on the size of the muscle were suggested as potential mechanisms that lead women to present with small muscle force and less resistance to fatigue^(10)^. It must be remembered that one of the limiting factors for the evaluation of the PGF measurement is due to advanced age and especially to the presence of Alzheimer's disease, factors that many times make its measurement non feasible.

The analysis of the biochemical tests showed reduced hemoglobin and hematocrit values. However, the levels of hemoglobin vary according to gender, age, smoking status, altitude of the residence, and physiological conditions. Additionally, the blood count components, such as hematocrit and hemoglobin, may have their values altered in some situations, such as parasitic infections, malignant neoplasms, heart failure, renal failure, inflammatory or infectious processes, hemorrhages, protein energy malnutrition, use of medications, and smoking, factors that are found in part of the population studied^(26)^.

Another proposed biochemical marker, with the objective of early detection of subclinical protein deficiencies, is albumin (transport protein present in plasma). Hypoalbuminemia was found in males for the age groups 60-65 years and 80-90 years. As to females, no reduced values were found. With aging, plasma albumin may suffer a slight decline, around 15 to 20%, in comparison with individuals of around 40 years of age, a ratio that also was observed in the evaluation of the sample, observing that one third of the population studied presented with reduced values both in men and in women^(27)^. Besides aging and the nutritional status of the individual, other factors may interfere in the absolute value of this parameter, such as liver diseases, congestive heart failure, nephrotic syndrome, enteropathies with loss of proteins, severe inflammatory process, liver failure, etc. Still, it is important to point out that the principal factor of low sensitivity of the albumin in the diagnosis of the acute phase of protein energy malnutrition, could be its relatively long biological half-life (± 20 days), and weeks may go by for a response to the variations in protein intake in diet^(28)^.

A study carried out in Minas Gerais by Coelho et al.^(29)^ with 197 elderly persons hospitalized, concluded that values of serum cholesterol >160mg/dL are identified as a protective factor for malnutrition, since results below this value may indicate reduction in lipoprotein levels and also of visceral proteins, and consequently, indicate malnutrition in the elderly. However, we must point out that hypocholesterolemia is also found in renal failure, liver failure, malabsorption, and also by the use of drugs of the statin class^(29)^. In the present study, it was noted that the majority of the male elderly individuals presented with reduced values of cholesterol when using drugs from the statin family.

A joint evaluation of the parameters studied suggests the final nutritional diagnosis, in some age groups, of sarcopenic obesity in the male group, which is associated with changes in body composition that occur in the aging process, including increased body fat and reduced muscle mass force. As per research done by Silva et al.^(30)^ with 288 men aged between 18 and 88 years, sarcopenic obesity occurs more frequently in males, since men, despite having more muscle mass, with aging experience greater loss relative to women, due to the decline of the growth hormone (GH), growth factor related to insulin (IGF-I), and testosterone. These data corroborate the result of the present study, since sarcopenic obesity was probably made evident in the male gender. Studies have further shown that the increase in body fat associated with sarcopenia represents a negative condition for health of elderly males, due to the increased risk of falls and fractures, decreased capacity to perform daily life activities, loss of independence, besides being associated with increased mortality^(30)^.

The biochemical parameters correlate positively with the nutritional status (except total cholesterol for the male gender). Thus, the elderly individuals that present with protein depletion identified in the anthropometric evaluation obtained reduced biochemical values, and in some cases, even below what is recommended, when compared to the elderly with no evidence of protein depletion, regardless of the gender.

In this way, the laboratory tests are a highly important complement for aiding in identification of the alterations that accompany aging and reflect the nutritional status, as well as the possible development of diseases.

## CONCLUSION

The results point to the need for other national anthropometric studies that allow a definition of reference standards directed at Brazilian elderly individuals, stratified by gender and by age range, since besides the difference between populations, some factors, such as inclusion and exclusion criteria and methodological characteristics limit the use of international standards and interfere in reliability of nutritional diagnosis.

Malnutrition in institutionalized elderly persons is a highly important topic in healthcare, and the present study presented the data from a Brazilian sample.

This research reinforced the need, and at the same time the difficulty in defining nutritional diagnosis in elderly institutionalized individuals, indicating the use of various anthropometric parameters, including TAPM and PGF, in order to obtain greater reliability in the nutritional diagnosis of this population. We further highlight that the results obtained with the use of each parameter should be evaluated with caution and jointly, in order to delineate the nutritional diagnosis of the elderly individual.

Also identified was the importance of the role of the dietician in caring for this population, and the need for more anthropometric studies that allow a definition of local reference standards, stratified by gender and age range, since, besides differences between populations, factors such as inclusion and exclusion criteria and methodological characteristics limit the use of international standards, interfering in the reliability of the nutritional diagnosis.

Due to the complexity of the factors involved in the evaluation and nutritional diagnosis of elderly persons, we point out the importance of working as a team, because a group of specialists with distinct academic training allows the integration and complementation of knowledge, enabling a more holistic vision, which will be very helpful in longevity and in quality of life of the elderly, especially when they are institutionalized.
